# Digital image correlation techniques for motion analysis and biomechanical characterization of plants

**DOI:** 10.3389/fpls.2023.1335445

**Published:** 2024-01-11

**Authors:** Max D. Mylo, Simon Poppinga

**Affiliations:** ^1^ Cluster of Excellence livMatS @ FIT – Freiburg Center for Interactive Materials and Bioinspired Technologies, University of Freiburg, Freiburg, Germany; ^2^ Department of Microsystems Engineering – IMTEK, University of Freiburg, Freiburg, Germany; ^3^ Botanical Garden, Department of Biology, Technical University of Darmstadt, Darmstadt, Germany

**Keywords:** complex movements, deformation mapping, digital volume correlation, motion characterization, strain analysis, photogrammetry

## Abstract

Temporally and spatially complex 3D deformation processes appear in plants in a variety of ways and are difficult to quantify in detail by classical cinematographic methods. Furthermore, many biomechanical test methods, e.g. regarding compression or tension, result in quasi-2D deformations of the tested structure, which are very time-consuming to analyze manually regarding strain fields. In materials testing, the contact-free optical 2D- or 3D-digital image correlation method (2D/3D-DIC) is common practice for similar tasks, but is still rather seldom used in the fundamental biological sciences. The present review aims to highlight the possibilities of 2D/3D-DIC for the plant sciences. The equipment, software, and preparative prerequisites are introduced in detail and advantages and disadvantages are discussed. In addition to the analysis of wood and trees, where DIC has been used since the 1990s, this is demonstrated by numerous recent approaches in the contexts of parasite-host attachment, cactus joint biomechanics, fruit peel impact resistance, and slow as well as fast movement phenomena in cones and traps of carnivorous plants. Despite some technical and preparative efforts, DIC is a very powerful tool for full-field 2D/3D displacement and strain analyses of plant structures, which is suitable for numerous in-depth research questions in the fields of plant biomechanics and morphogenesis.

## 1 Introduction

Plant biomechanics is a wide field of research which, in principle, studies the mechanical behavior and properties of plants. Combined with morphological and anatomical analyses, this field provides a central contribution to our understanding of form-structure-function relationships in plants (Speck and Speck, 2021). Plant biomechanics is a prime example of interdisciplinarity: beyond utilizing various biological methods, the discipline also makes use of methods from engineering, chemical, physical, mathematical, and material sciences (Geitmann et al., 2019). While conventional plant biomechanics research is lab-based, recently, there has been a push towards field- based ecological-biomechanical research, which has been bolstered by new generations of specialized outdoor equipment, like portable strain gauges and cameras (Bauer et al., 2020; Bauer and Poppinga, 2022; Ferry and Higham, 2022). Moreover, research in this field has advanced knowledge regarding developmental processes, biomaterials (wood, plant fibres) and their use in everyday products (furniture, clothing). Plant biomechanics is and will continue to be a cornerstone for biomimetics, as it offers an almost inexhaustible source of inspiration for developing bioinspired material systems (Speck and Speck, 2019).

Understanding the function and development of plant systems requires a thorough characterization of the mechanical properties of plants: ranging from annual herbaceous plants to century-old trees, and covering a magnitude of size classes, from cells and organelles, tissues and organs, to entire plants. Methods used in plant biomechanics range from classical analyses, such as mechanical testing (in tension, bending, torsion and/or compression), to more modern computer-based modelling and simulation approaches. However, biomechanical analyses of plant samples can only in exceptional cases be performed according to industrial standards (e.g. ISO or DIN), which are otherwise mandatory in conventional materials science and engineering approaches. Furthermore, they exhibit biological variance in shape, tissue composition, and further influencing factors such as water content. In addition, plant samples are usually multi-hierarchical compounds with soft transitions between the materials and structures involved, so that experimental techniques often cannot provide information about individual materials, but are limited to the entire “structural materials” (Wegst et al., 2015). Most conventional mechanical testing techniques, such as tensile or compression tests, are designed for homogeneous materials and only provide a mechanical characterization of the overall sample, but not of the local behavior. This gap can be bridged by combining classical mechanical testing with additional, more sophisticated approaches such as digital image correlation (DIC) analysis allowing for detailed displacement and strain analyses. In the following, we will first introduce the current state of the art in displacement and strain measurement, before looking in detail at the basics of the DIC method followed by a demonstration of its applicability to plant research, based on recent publications.

## 2 State-of-the-art methods for displacement and strain measurements in plant movements and morphogenesis

Prior research approaches incorporating complex 3D geometry analyses and displacement and strain analyses mostly deal with plant morphogenesis, i.e., organ and cell growth processes governed by biochemical and mechanical cues, and plant movements, which are driven by different means of actuation (Skotheim and Mahadevan, 2005; Eng and Sampathkumar, 2018; Bauer et al., 2021). Since 3D-DIC has already been used to quantify complex plant movements (see section 4.2 for examples), it is worthwhile concisely summarizing the different processes involved in such phenomena in the following.

The deformation processes of individual organs or organ parts are classically differentiated by whether they are nastic, meaning that the responses are morphologically predetermined and therefore always follow the same deformation patterns, or tropistic, where the responses depend on the direction of the stimuli (Hill and Findlay, 1981; Pietruszka and Lewicka, 2007). Exceptions are autonomous movements, which are not triggered by external stimuli (Migliaccio et al., 2013). Hydraulic motions are based on water displacement processes between cells and tissues and can be active or passive. Active hydraulic motion relies on metabolically costly processes like the opening and closing of ion channels resulting in reversible turgor change-induced and irreversible growth-based responses (Hill and Findlay, 1981; Dumais and Forterre, 2012; Harmer and Brooks, 2017). Passive hydraulics are, on the contrary, solely driven by humidity changes in the environment and include hygroscopic structures with dead cells, which can passively take up water and swell or lose water and shrink. Hygroscopic motions are known from many structures involved in diaspore dispersal like capsules and cones (Elbaum et al., 2008; Gallenmüller et al., 2018; Eger et al., 2022). Other examples from the passive-hydraulic motion group are structures that exhibit cohesion-force driven motions, like the annulus of the fern leptosporangium (Llorens et al., 2016). The speed of hydraulic motion is ultimately limited by the duration of the involved water flow processes between cells and tissues (the poroelastic time) and, therefore, depends on the dimension of the respective motile structure (Skotheim and Mahadevan, 2005). This dictates that hydraulically actuated large structures typically move slower in comparison to those which are small. Some plants overcome the poroelastic limitation by the storage and release of prestress, which is build up by foregoing movement processes (i.e., active and passive hydraulics). Elastic energy is thereby stored in the deformed plant structure and released ‘on demand’. The resulting motions are faster than hydraulics alone would achieve (Forterre, 2013). Famous examples from this group of elastic instabilities are the bursting fruits of the touch-me-not (*Impatiens* spp.) (Deegan, 2012) and of the dynamite tree (*Hura crepitans*) (Swaine and Beer, 1977). Plant motions can also be driven by combinations of the above-described hydraulic and elastic actuation principles. The snapping of the carnivorous Venus flytrap (*Dionaea muscipula*), for example, is driven by a combination of active hydraulics, which are initiated after prey has triggered the trap, and the release of prestress (Forterre et al., 2005; Sachse et al., 2020).

Although plant motion covers several timescales and includes numerous modes of deformation and actuation, most prior research covers only few parameters like general timescales (e.g., trapping durations in motile traps of carnivorous plants), distances travelled (e.g., in projectile seeds), or angular changes of the motile structure (e.g., seed scale bending in pinecones). The often much more complex temporal and spatial deformation sequences and concomitant developing strains on the motile structures are mostly only qualitatively described, if at all.

Strain gauges, which measure strains on objects via changes in electrical resistance due to object deformation, have been used in both laboratory- and field-based approaches, e.g., for continuous measurement of stem and fruit diameter (Link et al., 1998) and for probing the state of stress of wood in trees (Clair et al., 2013; Clair et al., 2019) with almost no temporal limitation. However, these devices deliver only limited local information of the structure under investigation. Larger strain fields can also be calculated by applying markers, e.g., dots, on the deforming structure and by subsequent computer-based manual or automated tracking (e.g., Jentzsch et al., 2022a).

de Reuille et al. (2015) introduced MorphoGraphX, a platform primarily used for the analysis and quantification of 4D live-imaged confocal microscopical image sets. It allows for high-resolution growth process analyses down to the sub-cellular resolution, which is a very powerful tool for in-depth investigations of developmental processes where complex multiscale interactions between genetic and mechanical processes are at place. The analyses can be quite labor-intensive depending on the method used for raw image acquisition and, for single-cell analyses, on the resulting signal for tissue segmentation. The raw data acquisition techniques are either limited to rather small samples [confocal laser scanning microscopy (CLSM), stereo scanning electron microscopy (Stereo-SEM), chemical force microscopy (CFM)] or are too time consuming for high temporal resolution data series [3D-scanner, magnetic resonance imaging (MRI)].

Kwiatkowska and Burian (2014) present a method which can be used for analyzing *in vivo* and comparing the 3D geometry of plant surfaces down to the cellular level with fairly low technical resources. Sequential replicas, i.e., series of moulds from a moving plant structure made in dental polymer and transferred to casts in epoxy resin can be subsequently analyzed, e.g., via SEM. With this technique, the original plant structure remains intact. The local geometrical changes (e.g., cellular swelling or shrinking processes) can then be computed and 3D reconstructed from the microscopical dataset. An example for this process is presented in Borowska-Wykręt et al. (2017), where the deformation of the hygroscopic bracts in *Helichrysum bracteatum* was investigated in detail. Forterre et al. (2005) present another example of strain quantification via mould comparison, in where they investigate the snapping of the carnivorous flytrap (*Dionaea muscipula*). The inner and outer trap lobe surfaces were moulded before and after trap closure and replicas were made with nail polish. For the follow-up microscopical analyses, structural trap features (hairs, glands) were tracked and the local strains computed. However, the required procedures are relatively time-consuming, especially when large numbers of intermediate steps are captured. For fast processes (like the Venus flytrap snapping), only start and end times can be comparatively analyzed. Furthermore, the moulding can probably lead to distortion of sensitive samples and, consequently, results.

## 3 Digital image correlation: basics and procedures

### 3.1 Digital image correlation technique

Digital image correlation (DIC) is a powerful technique typically used in experimental mechanics that allows non-contact measurement of full-field displacement and strain fields on the surface of structures. DIC operates by comparing digital images of an object taken at different times or under different loading conditions with respect to a reference state. DIC works principally by identifying small subsets of pixels within an image and tracks said pixels movement over time using image correlation algorithms. Strain fields can then be calculated through the relative displacement of neighboring subsets (Pan et al., 2009; Sutton et al., 2009).

DIC has its roots in the field of photogrammetry, which dates back to the mid-1800’s. It describes the use of photographs to make measurements and create maps and has been used extensively in cartography and surveying. Over the years, novel techniques have emerged and with the advent of digital cameras, photogrammetry entered the digital era in the 1980s (Schenk, 2005). It was during this period that the first DIC algorithms were developed (Peters and Ranson, 1982; Peters et al., 1983; Sutton et al., 1983; Chu et al., 1985), mainly for use in materials science to study the deformation and fracture behavior of various materials (such as metals, composites “or” polymers). Since then, DIC has been applied to a wide range of fields including civil engineering, geophysics “and” medical biomechanics (Sutton et al., 2009; Palanca et al., 2016). Over the years, DIC has undergone several improvements in terms of accuracy, speed “and” robustness. For example, advances in camera technology have enabled image capturing at higher resolutions and frame rates, while developments in computer processing power have allowed for faster and more complex algorithms to be implemented. The use of random intensity pattern (so-called “speckle patterns”) has become more widespread, which has led to improved tracking algorithms and more accurate measurements of displacement and strain fields (compared to the manual marker tracking mentioned in section 2). In addition, the required hardware became cheaper and readily available, so that DIC became an increasingly widespread measurement technique (Pan, 2018).

The advantages of DIC are that it is a non-contact and non-invasive technique due to its optical imaging and that it has no temporal resolution limitation. The temporal resolution is solely determined by the sampling rate of the cameras used. This makes DIC suitable for both time-lapse recordings of relatively slow movements that take place over hours to days (e.g., hygroscopic plant motions; Correa et al., 2020) and high-speed recordings of rapid movements that take place in fractions of a second (e.g., the snapping of carnivorous plants or rapid seed dispersal; Siebert et al., 2007; Reu and Miller, 2008; Beberniss and Ehrhardt, 2017; Sachse et al., 2020). The spatial resolution of the method is highly dependent on the cameras and lenses used, as well as on the contrast of the surface examined, allowing characterization from the nanoscale (objects in the micrometer to millimeter range; Knauss et al., 2003; Berfield et al., 2007) to the macroscale (objects of several meters; Yoneyama et al., 2007). **Table 1** summarizes the most important factors influencing the spatial resolution of a DIC measurement. Due to technical advances, today’s evaluation PCs can analyze a large number of subsets and thus contribute to a high local resolution. However, depending on the number of deformation images to be processed, computation time may still be a factor when analyzing 3D-DIC datasets.

**Table 1:** Overview of the factors and parameters that influence and determine the spatial resolution of DIC measurements (2D and 3D).

Factors influencing spatial resolution	Description
**Camera sensor size**	The more pixels the camera sensor has, the more subsets of the same pixel size can be analyzed on the sample surface.
**Camera sensor quality**	The quality of the camera sensor affects image noise, dynamic range, and sensitivity, with better sensors giving cleaner images.
**Lens quality**	The lens quality affects the sharpness and distortion of the images.
**Lens aperture**	Closing the aperture further increases the depth of field and therefore resolution, especially for highly curved samples or large movements. However, if the aperture is closed too far, there is a risk of aliasing, which reduces accuracy.
**Object surface contrast**	High contrast patterns increase the accuracy and quality of subset correlation, with a 50:50 distribution of black and white pixels being optimal.
**Speckle size**	The size of the speckles must match the object and the camera system. About 3-5 speckles per subset should be visible, and the size of the subsets should be as homogeneous as possible.
**Lightning conditions**	Uneven lighting or glare can create shadows and reflections, making it difficult to accurately track subsets.
**Interpolation method**	Advanced interpolation methods are used to estimate displacements with sub-pixel accuracy.
**Subset size**	The subset size is the most important parameter in determining the spatial resolution of DIC. Smaller subsets provide better spatial resolution, but may result in decreased accuracy.
**Subset distance**	The distance or overlap of adjacent subsets is another important parameter that determines the spatial resolution and should be selected depending on the subset size. Smaller distances increase the resolution but also the computation time.

Furthermore, the DIC method is not limited to classical digital cameras (mostly CCD sensors). Rather, it can be applied to image series from light microscopes, infrared cameras, fluorescence microscopes, electron microscopes (SEM, TEM, STEM), scanning probe microscopes (AFM, STM) “or” laser scanning confocal microscopes (LSCM), as well as methods of 3D imaging (CT, MRI for digital volume correlation; see section 3.6) (Pan et al., 2009; Pan, 2018).

### 3.2 2D-DIC vs. 3D-DIC

**Figure 1:**
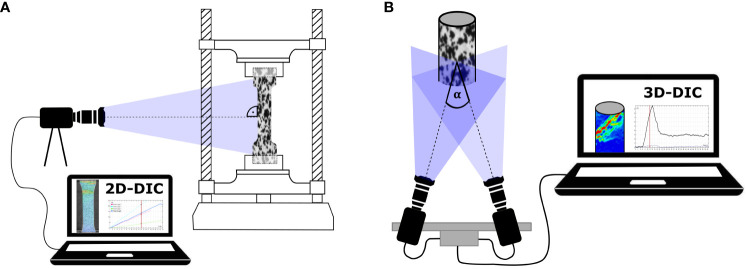
Schematic representation of DIC setups. **(A)** 2D-DIC setup with one camera oriented perpendicular to the surface of a speckled dog bone-shaped sample clamped in a tensile testing machine for the displacement and strain analysis. **(B)** 3D-DIC setup (stereo-DIC) with two synchronized cameras capturing the surface of a speckled cylinder from different camera positions, resulting in a stereo angle α of approximately 20-30°.

There are two main types of DIC, namely 2D-DIC and 3D-DIC (also known as stereo-DIC). 2D-DIC is used when the surface for analysis has little or no curvature and is predominantly deformed in plane. 3D DIC is used for objects with a non-planar surface and/or to analyze out-of-plane deformation (Sutton et al., 2008). 2D-DIC uses images from a single camera whose sensor is aligned parallel to the sample surface and does not require calibration of the measurement field (**Figure 1A**). In contrast, 3D-DIC analysis is based on stereo images captured by two synchronized cameras with identical sensors. These sensors are aligned at a stereo angle to the object of approximately 20-30°, thus capturing the surface from different viewing angles (**Figure 1B**). Here, the measurement volume must be calibrated before the actual experiment (Pan, 2018). In both systems, the resolution of the cameras is a crucial determinant of the resulting spatial resolution of the measurements. It should be noted that especially for 3D-DIC, the depth of focus is an important factor because data processing requires completely sharp images. This requirement impedes, to a certain extent, the analysis of small structures, which require high optical magnification, and/or spatially very complex curved structures.

Depending on the surface of the sample and its expected movement and deformation, it must initially be decided whether a cost-effective and time-saving 2D-DIC analysis is sufficient or whether a stereo camera setup with time-consuming calibration is required for 3D-DIC analysis. **Table 2** provides an overview of open source and commercially available 2D-DIC and 3D-DIC software. In addition to the DIC analysis options, some of the software listed also offer the following: digital volume correlation, acquisition with more than two cameras (multi-DIC; see section 3.6), matching with finite element (FE) simulations or other features. For details, visit the respective homepages or contact the companies.

**Table 2:** Overview of existing 2D and 3D-DIC software and their licenses.

Software	Company/ Programming language	2D or 3D-DIC	License
2D-ALDIC	MATLAB	2D	Open-Source
ADIC2D/ADIC3D	MATLAB	2D & 3D	Open-Source
DICe	C++	2D & 3D	Open-Source
Digital Image Correlation and Tracking	MATLAB	2D	Open-Source
DuoDIC	MATLAB	2D	Open-Source
EikoTwin DIC	EikoSim	2D & 3D	Commercial
GOM Correlate	GOM Metrology (Zeiss)	2D	Freeware^1^
GOM Correlate Pro^2^	GOM Metrology (Zeiss)	2D & 3D	Commercial
iCorrVision	Python	2D & 3D	Open-Source
Istra4D	Dantec Dynamics	2D & 3D	Commercial
MatchID	MatchID	2D & 3D	Commercial
Ncorr	MATLAB/C++	2D	Open-Source
py2DIC	Python	2D	Open-Source
pydic	Python	2D	Open-Source
pyxel	Python	2D	Open-Source
StrainMaster	LaVision	2D & 3D	Commercial
Tema	Image Systems	2D & 3D	Commercial
UFreckles	MATLAB	2D & 3D	Open-Source
VIC-2D/VIC-3D^3^	Correlated Solutions	2D & 3D	Commercial
YaDICs	Linux	2D	Open-Source

^1^Registration required.

^2^Formerly known as ARAMIS.

^3^Low-cost academic system available (VIC-EDU).

### 3.3 System calibration

Calibration is crucial in 3D-DIC measurements as it establishes a relationship between the digital images and the physical dimensions in the real world by determining both intrinsic (i.e., internal) and extrinsic (i.e., external) parameters. The intrinsic parameters include the focal length and the lens distortion of the cameras. The extrinsic parameters are related to the camera’s position and orientation during image acquisition (Sutton et al., 2008). To acquire accurate measurements there must be a calibration target with known geometric features. The target should possess distinctive and easily identifiable markers or patterns that can be tracked in the images (e.g., checkerboard pattern, coded dot pattern “or” random speckle pattern). Additionally, the calibration target should ideally cover the same field of view as the subsequent measurements to ensure consistency and to maximize distortion corrections (Sutton et al., 2009; Chen and Pan, 2020). Calibration objects are usually included with the purchase of a commercial DIC system, but there are also freely available pattern generators that allow you to print or machine your own target at low cost (Correlated Solutions, Inc, 2023; MVTec Software GmbH, 2023).

During calibration, a series of stereo images of the target are captured from different orientations and positions. By analyzing the known dimensions of the calibration target in the images, the internal and external parameters can be computed (Sutton et al., 2008). In the end of calibration, it generates a calibration matrix that relates the pixel coordinates of the images to physical displacements. The calibration information includes both intrinsic and extrinsic parameters and is often stored in a calibration output file. This information is then used during the subsequent measurement phase to accurately determine displacements and deformations on the analyzed surface.

### 3.4 Sample preparation

As discussed above, the choice of camera system depends, among other factors, on the nature of the sample surface (2D-DIC for flat surfaces and 3D-DIC for curved/structured surfaces). However, not all surfaces are suitable for stereo camera imaging. For 3D-DIC analysis, all areas of the surface must be visible in both cameras at all times, otherwise this will cause gaps in the subsequent surface detection. Therefore, if the sample is highly curved or has pronounced edges or protrusions, the surface or parts of the surface cannot be tracked and analyzed.

**Figure 2:**
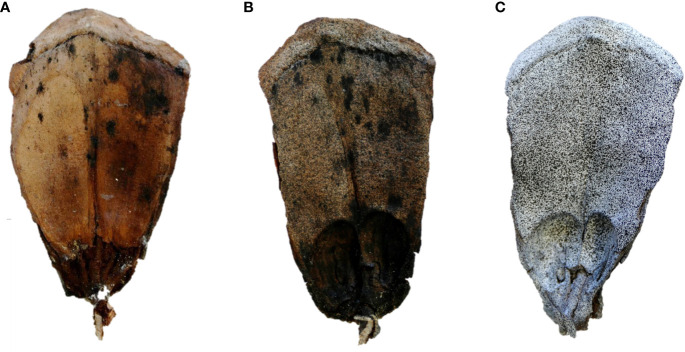
Surface preparation for DIC analysis using the example of the upper surfaces of *Pinus lambertiana* cone scales. **(A)** An untreated scale showing only the natural color contrasts. **(B)** A scale sprayed with a random pattern of black speckles. However, due to the natural brownish color of the scale, there is little brightness contrast. **(C)** A scale primed with white paint before being sprayed with a black speckled pattern. This provides the best possible brightness contrast, which is a prerequisite for high-resolution DIC analysis.

Once a suitable sample has been identified, preparing the surface is the next crucial step in DIC experiments. To obtain reliable and accurate results, the surface must contain a random speckle pattern to calculate the correlation (**Figure 2**). Some plant samples naturally have a reasonably good pattern on their surface (leaf patterns, wood samples, bark, etc.), but this not usually the case (Gauvin et al., 2014; Dong and Pan, 2017; **Figure 2A**). The pattern quality has a strong influence on the DIC results, therefore it is important to always test whether an artificial pattern would be advantageous.

A speckle pattern is considered high quality when it adheres to four factors: (1) The grey values of the surface must have as high a contrast as possible (preferably black speckles on a white background or vice versa). (2) The patterns must not be periodic, but random, to make false correlations as unlikely as possible. (3) The size of the speckles and their density over the surface should be kept constant, with individual speckles being at least 3 pixels in size. (4) The applied pattern should not change the mechanical properties of the sample and should adhere optimally to the sample even under large deformations (Dong and Pan, 2017; Pan, 2018). For an in-depth overview of the perfect size, contrast, edge sharpness “and” aliasing effects of the patterns, we recommend Reu’s four-part ‘All about speckles’ collection (Reu, 2014a; Reu, 2014b; Reu, 2015a; Reu, 2015b).

For glossy surfaces, use an anti-glare spray before applying a speckle pattern to achieve uniform illumination of the object. In the case of heterogeneous surfaces or surfaces of medium brightness, applying a prime coat can enhance the resulting contrast (**Figures 2B, C**). However, for biological analyses (especially those involving evaporation mechanisms) it is important to consider that extensively treating the surface may result in reduced evaporation and, therefore, slower or altered movement patterns (Correa et al., 2020). There are various methods of applying a random speckle pattern, with a general distinction between non-destructive and destructive types, depending on the effect on the integrity of the sample (Dong and Pan, 2017). Since the use of destructive methods, such as surface notching, is associated with physiological or physical disturbance for most biological samples, we will focus on non-destructive methods in the following section. Among the non-destructive methods commonly used in biomechanical analyses, spraying paint (also possible with paint dipped combs or brushes, where the bristles vibrate and spray paint by deflection) and airbrushing are the most commonly used. Both methods are suitable for macroscopic to microscopic samples and are characterized by their relatively low costs, minimal time requirements, ease of handling and variability of speckle sizes (Dong and Pan, 2017). The latter can be controlled by selecting the appropriate spray head, adjusting the distance to the sample, (air) pressure, and the viscosity of the paint (Lionello and Cristofolini, 2014). In addition, stamping, spin coating, focused ion beam milling “and” other techniques are used, particularly for smaller samples. Although there is a wealth of literature on pattern quality assessment (Lecompte et al., 2006; Yaofeng and Pang, 2007; Pan et al., 2008; Pan et al., 2010; Hua et al., 2011; Lionello and Cristofolini, 2014), practical experience with biological samples and their surfaces has shown that a degree of trial and error is often unavoidable. Some of the available DIC software include functions for quantifying the quality of surface patterns.

### 3.5 Image correlation and data processing

**Figure 3:**
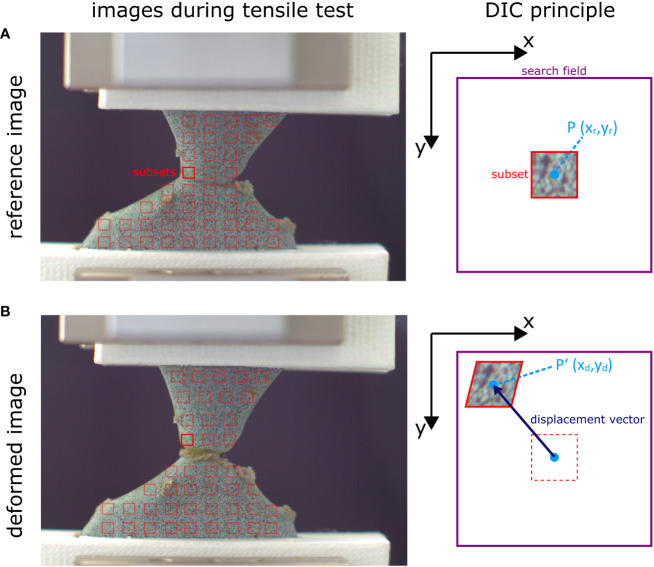
Example of subset displacement of a cactus junction under tensile loading. The surface of interest of the reference image **(A)** is divided into smaller subset (red squares) which are tracked over the stack of deformation images **(B)**. The grey-value distribution of each subset is searched within a defined field (purple square). This is illustrated on the right for the bright red subset. Depending on the highest correlation value, the subset center P is displaced to its new location P’, resulting in a displacement vector. By calculating the vectors of all the subsets, vector fields and strain patterns (via the relative displacement of neighboring subsets) can be generated. Please note that for better visualization, the distance between subsets in this figure is larger than what is usual in most analyses. Often, adjacent subsets overlap to provide the highest possible spatial resolution. Figure modified from Mylo (2022).

The data base for DIC analysis consist of image series from one (for 2D-DIC) or two (for 3D-DIC) cameras. These image series cover the sample surface of interest in the undeformed state (reference image; **Figure 3A**) and during deformation or motion (deformed images; **Figure 3B**). These images are divided into a series of small areas, usually referred to as subsets, facets “or” windows, which serve as the basic units for correlation analysis. Within each subset, the greyscale intensity distributions of the two images are compared using correlation algorithms. The correlation process involves shifting, rotating “and” deforming one subset between two images. This is done by calculating correlation coefficients or similarity measures (such as cross-correlations or sum of squared differences), between the grey value of the corresponding pixels in the subsets (Pan, 2011). The location with the highest correlation indicates the shift that has occurred between the two images, resulting in a deformation vector of the subset midpoint (Pan et al., 2009; Sutton et al., 2009; **Figure 3**). The calculation for correlation may vary slightly from software to software, although some software allow the user to select the calculation method in the expert settings. Depending on the DIC software used, the pixel size of the subsets, the distance between two adjusting subset centers “and” the search area for correlation must be defined. The values which are most appropriate depend heavily on the pixel resolution of the camera, the speckle pattern, and the expected motion of the sample. The size of the subsets determines the spatial resolution of the measurement. However, if they are too small, the likelihood of erroneous correlations increases, so there is a trade-off between spatial resolution and measurement accuracy.

**Figure 4:**
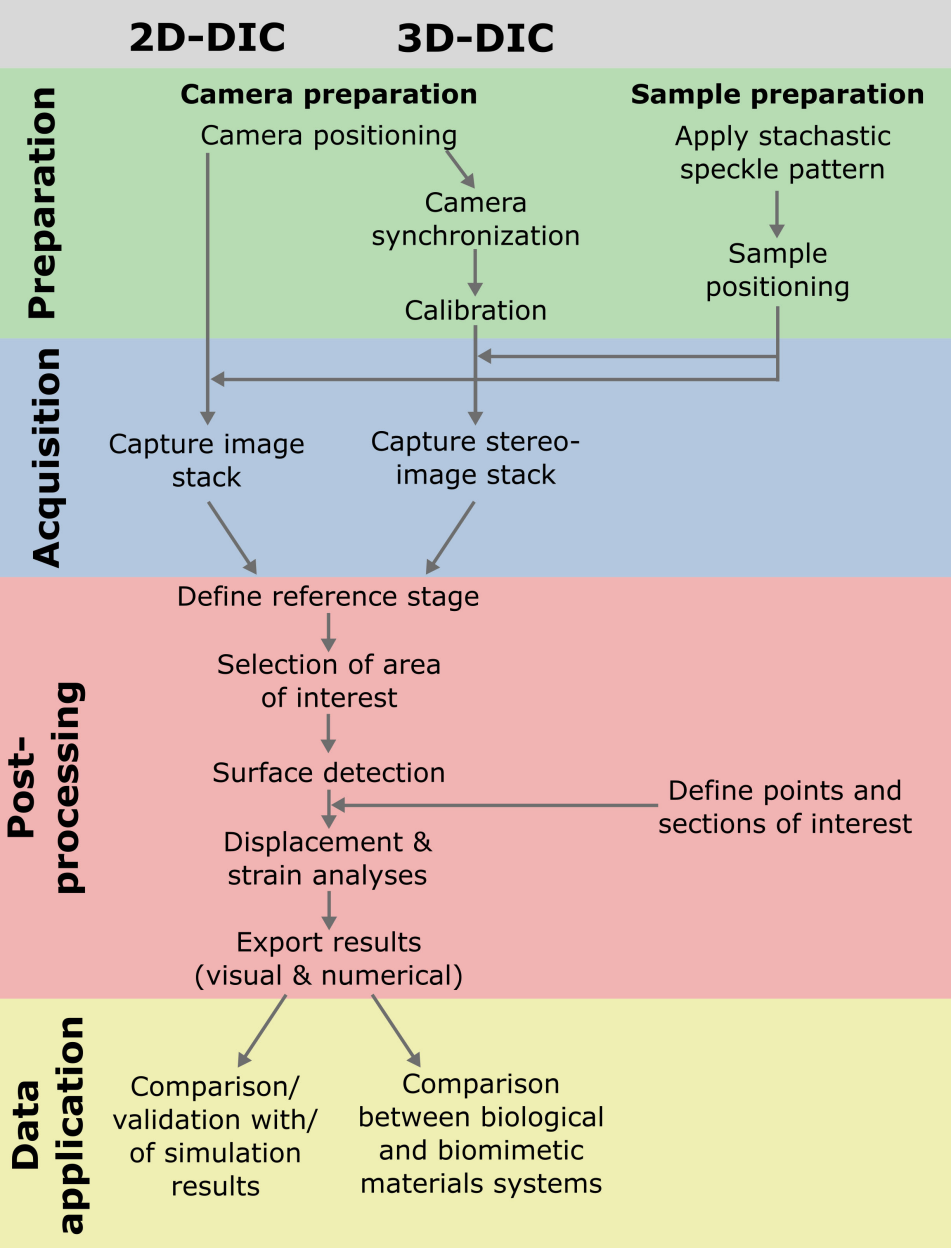
Example of workflows of 2D and 3D-DIC experiments from preparation to acquisition, post-processing and possible application of the data (here in the context of biomimetics).

The correlation process is repeated for all subsets across the surface of interest, resulting in a displacement vector map. To increase accuracy, interpolation techniques can be used to estimate sub-pixel displacements (Pan et al., 2006; Sutton et al., 2009). A strain field can be calculated from the displacement vectors via the relative relationships of adjacent subsets. Acceleration and strain rates can be calculated from the time intervals between two images. By performing these correlation steps over the entirety of the selected region of interest of the recorded deformation images, the surface movement or deformation pattern can be visualized over time. In addition to displaying movement or deformation patterns, many of the software packages available have the ability to analyze and quantify individual points and sections in detail. The resulting displacement and strain data can be used for material or motion characterization, quantitative comparison of structures, or as a basis or validation for FE simulations. **Figure 4** shows an example of DIC workflow from sample and setup preparation to data application.

### 3.6 Multi-DIC, DVC and further techniques

Despite all of its advantages, DIC also comes with some limitations that require more sophisticated camera setups. For example, although a stereo camera setup can provide a 3D visualization of the surface, it is always limited to the area of the sample visible to the cameras. Part of the motion and deformation remains unresolved, providing limited information, especially for multi-material systems or very heterogeneous materials, as is often the case with plant samples (Sachse et al., 2020; Durak et al., 2022b; Mylo et al., 2022b). One way to overcome this is to use a multi-view DIC system, where one or more (stereo) camera setups are installed around the sample to provide additional viewing angles (Chen et al., 2013; Patil et al., 2017; Janeliukstis and Chen, 2021). For 360° DIC analysis, it is also possible to position several cameras in a circle around the sample and then combine two adjacent cameras for 3D-DIC analysis and merge the individual resulting surfaces (Solav et al., 2018; Srivastava and Baqersad, 2019; Genovese et al., 2021; Li et al., 2023). Another possibility would be to rotate the cameras around the sample (Genovese et al., 2016). However, the authors are not aware of any work that has used this kind of laborious setup for analyzing plant systems. Another solution for generating 360° images with only two cameras, would be to place the sample in front of the cameras on a rotary stage and rotate it in defined degree steps to acquire and stitch 3D-DIC images from different perspectives. However, the time offset during the rotations would prevent investigations of comparatively fast deformations. Additional approaches use mirrors placed behind the sample to capture a second perspective (Chen and Pan, 2019b; Pan and Chen, 2019; Chen et al., 2020; Chen and Pan, 2022). However, as most DIC software assumes a pinhole camera system with a straight optical path, calibrating the system with mirror images would require altering the calibration algorithms. Similarly, when analyzing samples immersed in water/liquids or samples behind glass (e.g., in a warming cabinet with a window), most 3D-DIC systems are not able to take the different refractive indices of the different media in their calibration algorithms into account. The authors are only aware of the VIC-3D software from Correlated Solutions with the Variable Ray Origin (VRO) feature that provides a solution to this challenge, allowing measurements through glass or liquids (Correlated Solutions, Inc, 2021).

Single camera setups can be specially adapted for 3D DIC analysis. Prisms (Lee et al., 1999; Lee and Kweon, 2000; Dan et al., 2019), diffraction gratings (Pan et al., 2015; Pan et al., 2016), mirrors (Zhang and Tsui, 1998; Gluckman and Nayar, 1999; Yu and Pan, 2017b) or band-pass filters (Yu and Pan, 2017a; Wang et al., 2018b) are used to separate the individual wavelengths of light or to reflect its path, providing multi-perspectivity that mimics the viewing angles of a stereo camera system, and, depending on the setup, even allows 360° scans (Chen and Pan, 2019a). The advantage of these systems is that a single camera is sufficient for 3D images, eliminating the need to synchronize the cameras. However, these setups require additional optical components and adapted DIC algorithms, making them not trivial to operate for the common user (Pan et al., 2018). In this article, we will therefore limit the discussion of 3D-DIC systems to the classic stereo camera setups.

Another limitation of digital image correlation is that it only measures surface motion and strain. To generate full volume information, its sister technique, digital volume correlation (DVC), is required (Bay, 2008; Roberts et al., 2014; Buljac et al., 2018). The input for DVC are volume data sets at different time points or loading conditions, for example *in-situ* tests recorded by computed tomography (CT; Bay et al., 1999; Boulanaache et al., 2020) or magnetic resonance imaging (MRI; Tavana et al., 2020; Tavana et al., 2021). The underlying correlation calculations are similar to those used in DIC, but refer to 3D voxels rather than 2D subsets. With the ability to visualize local strain in the sample volume, DVC is a powerful tool for detecting inhomogeneities and defects that occur under load in (composite) materials. This method also requires good brightness and contrast in the volume to be analyzed. This, together with the more sophisticated technology required, makes the range of applications and availability of data less widespread than for DIC. However, due to its great potential and expected technical advances, DVC will most presumably be increasingly used in materials science in the coming years (Sutton and Hild, 2015), including for the analysis of plant and bioinspired material systems. **Table 3** provides a comparative summary of the features, requirements and limitations of surface image correlation (2D and 3D-DIC) and volume image correlation (DVC).

**Table 3:** Overview of the features, capabilities, requirements and limitations of 2D-DIC, 3D-DIC and DVC systems.

Feature	2D-DIC	3D-DIC	DVC
**Measuring geometry**	surface	surface	volume
**Measuring dimensions**	2D	3D	3D
**Capture device**	one camera	(at least) two cameras	volume scan
**Sample requirement**	surface speckle pattern	surface speckle pattern	volume speckle pattern
**Measuring limitation**	in-plane analysis of planar surfaces	out-of-plane analysis of irregular surfaces	any movement/deformation of arbitrary samples
**Calibration**	not required	required	not required
**Sample dimension**	limited by camera & lens	limited by camera & lens plus size of calibration plate	limited by maximum scanning volume
**Temporal resolution**	limited by camera frame rate	limited by camera frame rate	limited by scan duration

## 4 Recent examples for digital image correlation in plant sciences

### 4.1 DIC for plant biomechanical analyses

The following sections concisely summarize published articles where DIC methods were successfully applied to solve relevant scientific questions.

#### 4.1.1 Parasite-host attachment

**Figure 5:**
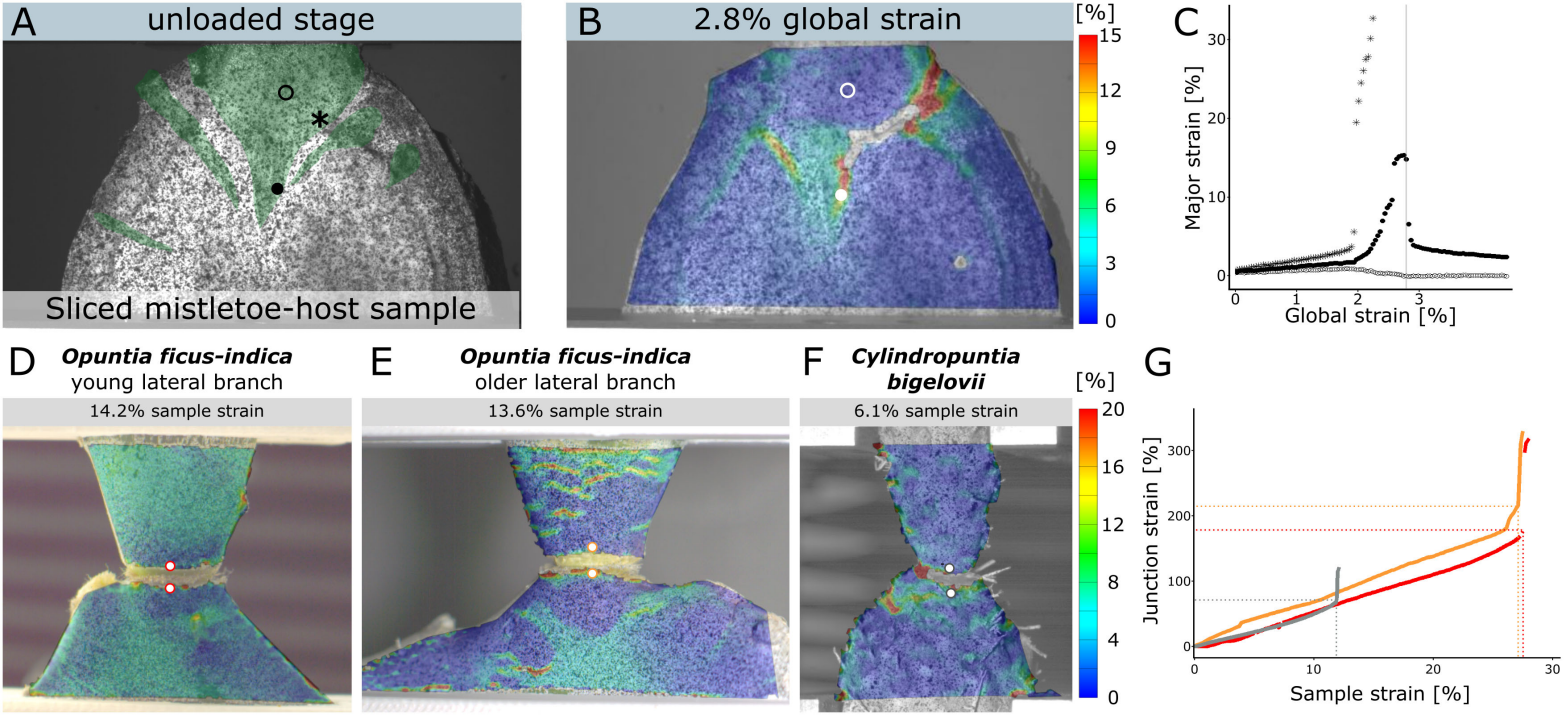
2D-DIC measurements of European mistletoe (*Viscum album*) and cacti (*Opuntia ficus-indica* and *Cylindropuntia bigelovii*) samples. **(A–C)** Strain analysis of a sliced section through a mistletoe-host attachment site under tensile loading (modified from Mylo et al., 2022a). **(A)** Sample with applied speckle pattern in unloaded state (reference image). The mistletoe tissue (haustorium with sinkers) is artificially colored green. Three points (empty circle in the center of the sinker, asterisk at the mistletoe/host interface and filled circle at the tip of the sinker) are marked for local strain quantification. **(B)** Local major strain distribution at 2.8% strain of the entire sample, with failure initiating along the interface. **(C)** Local strain of the three points during tensile testing, with the vertical grey line marking the strain state of **(B)**. **(D–G)** Strain analysis of branch-branch junctions in cacti under tensile loading (modified from Mylo et al., 2022b). Strain distribution of a young junction of *Opuntia ficus-indica* **(D)**, an older junction of *O. ficus-indica* **(E)** and a junction of *Cylindropuntia bigelovii* **(F)** at about half the strain rate that led to failure. The strain scale applies to all cactus subfigures. **(G)** shows the junction strains (as the strain between the selected points above and below the junction) of the three samples as a function of the total sample strain (as the strain of the tensile testing clamps comprising the junctions and parts of the adjacent branches).

The evergreen European mistletoe (*Viscum album*) is one of the most common and best-known parasitic plants in Central Europe. It grows on the branches of its host trees, from which it extracts water and dissolved nutrients through the haustorium (“shoot-root mosaic” organ; Teixeira-Costa, 2021), which consists of several sinkers (Mylo et al., 2021b). Despite its size of up to more than two meters in diameter and the associated mechanical stresses, its attachment to the host is very robust (Mylo and Speck, 2023), such that a naturally occurring interface failure has never been reported. To characterize the mechanical behavior of the interface, Mylo et al. (2022a) performed uniaxial tensile tests on intact and sliced samples through the mistletoe-host attachment site. A speckle pattern was sprayed onto one of the resulting surfaces of the sliced samples to analyze local strains by 2D-DIC under tensile loading (**Figure 5A**). The resulting strain patterns revealed that the largest strain values occur directly along the interface between mistletoe and host (**Figure 5B**). Analysis of a single point at the interface (marked with an asterisk) indicates that strains greater than 30% are required to initiate failure along the mistletoe-host interface. Hardly any strain was measured in the inner parts of the main sinker (marked with an empty circle). The maximum strain in the tip of the sinker exceeded 15%, but relaxed almost completely after the sample failed, suggesting loading in the elastic range (marked with a filled circle; **Figure 5C**). Gaps in the surface detection along the fracture became visible, which can be explained by the very large strains and fracture running through individual subsets (**Figure 5B**). The strain analyses on the mistletoe-host samples are a good illustration on why a semi-quantitative comparison of strain patterns should be combined with a purely quantitative analysis of individual points [or sections, see also Mylo et al. (2022a)] when evaluating DIC tests. Particularly in the case of complex strain patterns, the choice of measurement points must be carefully considered, as local data points may vary markedly.

#### 4.1.2 Cactus junctions

Members of the Cacti subfamily Opuntioideae are characterized by a number of xerophytic adaptations (Gibson and Nobel, 1986; Rebman and Pinkava, 2001; Mylo et al., 2020). Some Opuntioids, such as *Cylindropuntia bigelovii*, are known to shed their lateral branches under slight mechanical forces. The detached branches can adhere to the fur of mammals with their retrorse-shaped barbs spines, be carried away, form roots and grow into new individuals (Bobich and Nobel, 2001). Other species, such as *Opuntia ficus-indica*, stiffen their branch-branch connections in early stages by periderm attachment and rapid proliferation, allowing them to reach a tree-like habit (Gibson and Nobel, 1986; Mylo et al., 2021a). Mylo et al. (2022b) have mechanically characterized the lateral branch-branch connections using uniaxial tensile tests. 2D-DIC recordings of the samples were used to analyze how much the junction is elongated under load and how much of the forces are taken up by the adjoining branches. The strain patterns revealed that the strains in *C. bigelovii* (**Figure 5F**) are mainly concentrated in the area directly around the junction, whereas in *O. ficus-indica*, for both the younger (**Figure 5D**) and the older (**Figure 5E**) samples, the adjacent branches also take up strain. This contributes to the fact that the total elongation of the sample at failure is about two to three times higher for *O. ficus-indica* than for branch-branch junction samples of *C. bigelovii* (Mylo et al., 2022b). What is immediately noticeable in the data is the loss of surface detection along the junction. The strain here is up to 200%, which makes correlation of the subsets impossible after a certain point. As a workaround, two points were selected above and below the junction whose pattern was detected over the entire tensile test. The strain between these two points was defined as the junction strain and plotted against the total strain of the samples (**Figure 5G**).

The samples analyzed are well suited to demonstrate a limitation of the DIC technique: The irregular surface of the samples would technically require a 3D analysis. However, with the undulating surface of the *C. bigelovii* branches, it was not possible to have all points of the surface in the image with both cameras using a stereo camera setup, which would lead to large gaps in a 3D-DIC surface coverage. A 2D-DIC analysis circumvents this problem, but the data should be evaluated and compared with the caveat that they have measurement errors caused by the lack of depth information.

#### 4.1.3 Fruit peels

**Figure 6:**
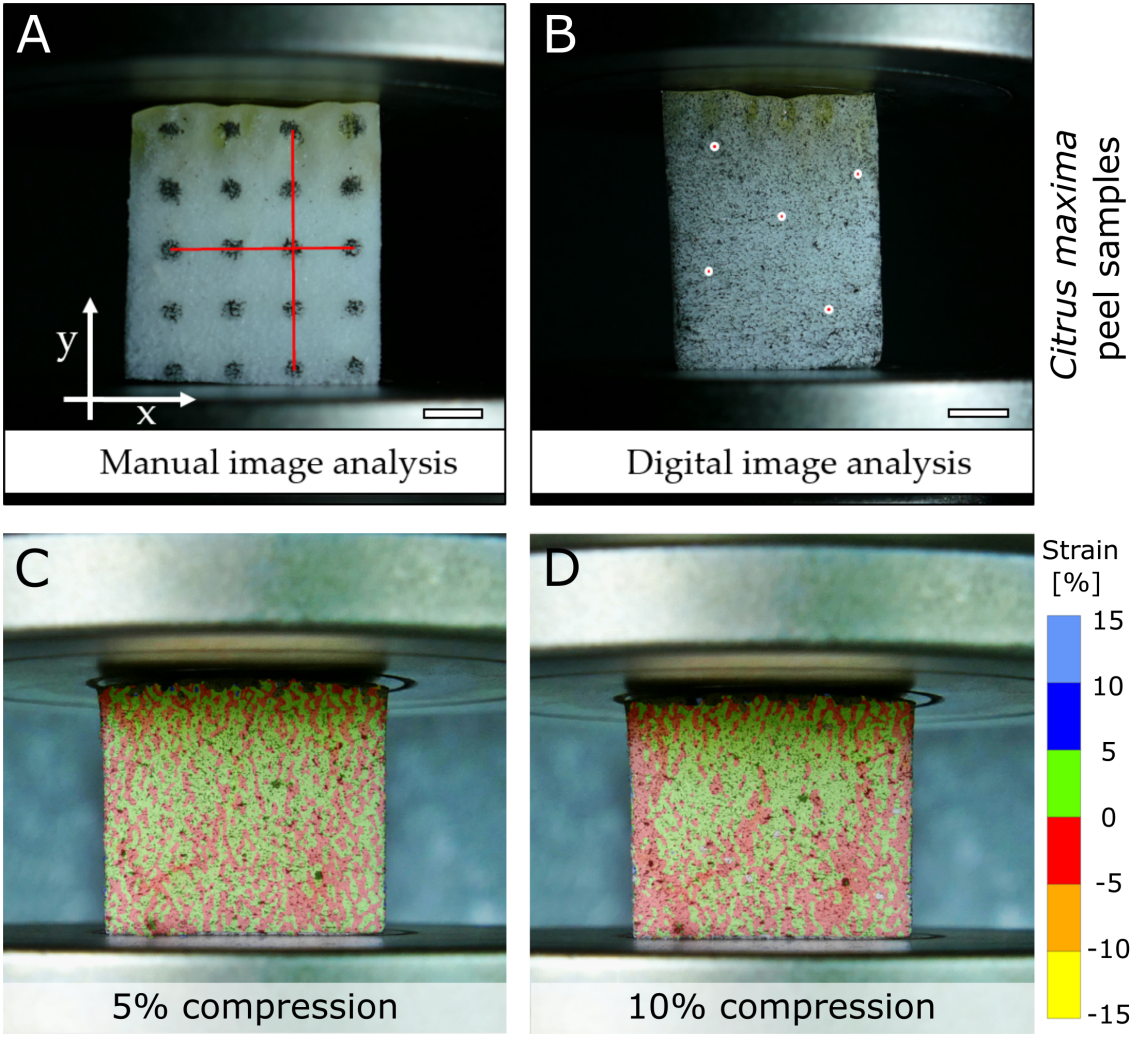
Comparative analysis of a manual marker tracking and DIC approach to measure the Poisson’s ratio of a citrus peel (*Citrus maxima*) under compressive loading. For manual tracking, a defined dot pattern was applied to the sample and analyzed **(A)**. For the DIC measurement, a stochastic speckle pattern was applied, with five points selected for further analysis **(B)**. The scale bars equal 5 mm. **(C&D)** show the patterns of strain in lateral direction of the DIC sample at 5% compression **(C)** and at 10% compression **(D)**. Figure modified from Jentzsch et al., 2022a.

The peel of many citrus fruits has particularly good damping properties, so that they can survive a drop from a height of several meters without causing major damage that would facilitate the penetration of pathogens (Thielen et al., 2013a; Thielen et al., 2013b; Jentzsch et al., 2022b). To analyze their mechanical properties, Jentzsch et al. (2022a) carried out compression tests on the peel, consisting of mesocarp, endocarp “and” exocarp. To measure Poisson’s ratio, which describes the ratio of deformation of a sample under perpendicular force, of the peel of five different *Citrus* species, a manual marker tracking approach (**Figure 6A**) and a 2D-DIC approach (**Figure 6B**) were compared. No significant differences were found between the results of the two techniques, with Poisson’s ratios being around 0. However, it was noticeable that the DIC method showed a much smaller scatter of data within the analyzed samples of a species and can therefore be considered as a more reliable measurement. The strains of the different species showed different patterns, with negative lateral strains in the outer areas of *Citrus maxima* and positive strains in the center of the quadratic samples (**Figures 6C, D**). The DIC evaluations were limited to a total sample compression of 20%, as the surface detection exhibited larger gaps at higher compressions.

### 4.2 DIC for plant motion analyses

#### 4.2.1 Opening and closing of pine cones

The cones of pines (*Pinus* spp.) are characterized by numerous seed scales arranged in a Fibonacci pattern around the cone axis. To facilitate seed shedding under optimal conditions for wind dispersal, the scales bend downwards when it is dry (thereby releasing the seeds) and upwards under wet environmental conditions (thereby keeping and protecting the seeds). The reversible, passive-hydraulic movements are due to the swelling and shrinking of a sclereid layer within each scale, which actuates the bending deformation, whereas other tissues have different functions, e.g., sclerenchyma fibers acting as a mechanical resistance layer (Dawson et al., 1997; Eger et al., 2022).

**Figure 7:**
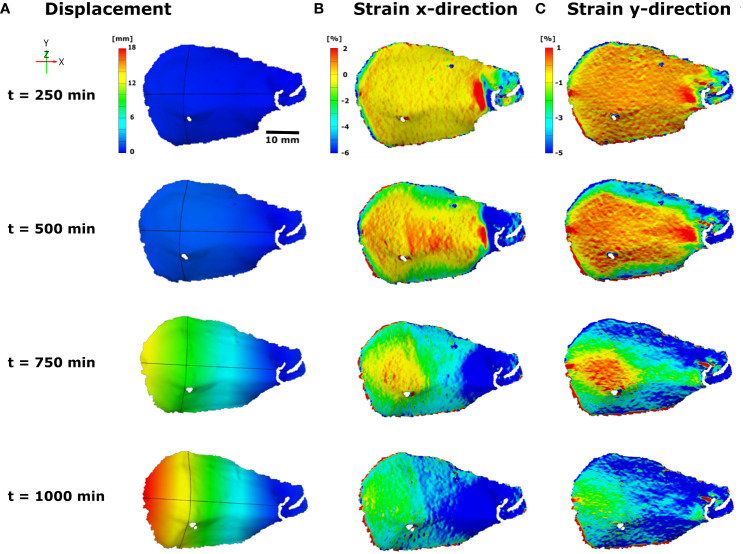
3D-DIC displacement and strain measurements on the abaxial seed scale surface of the Bhutan pine (*Pinus wallichiana*) during desiccation-driven bending deformation, recorded via time-lapse. The coordinate, timescale [min], and color schemes for displacement [mm] and strain ([%], x and y-direction) values are indicated. **(A)** In the displacement analysis the synchronous displacement of the lateral flaps and subsequent longitudinal bending of the scale can be seen, with the scale base (which is naturally attached to the cone axis) not moving at all and the scale tip moving the most. **(B)** The increasing strain in x-direction over the course of time most presumably is responsible for the longitudinal bending, whereas **(C)** the increase in strain in y-direction dictates the “flattening” of the scale. Figure modified from Correa et al. (2020).

Correa et al. (2020) observed a distinct two-phase motion behavior of initially wet Bhutan pine (*Pinus wallichiana*) seed scales during desiccation, during which first the transverse curvature of the scale changes, followed by its pronounced longitudinal bending deformation. They speculate that the transversal scale curvature in the wet state, which corresponds to the closed cone when all scales are bent upwards, increases the moment of inertia and thereby causes a higher flexural stiffness of each scale, enhancing the mechanical protection from seed predation (Elliott, 1974). The observed multi-phase hygroscopic motion prompted deeper investigations of the deformation behavior of the scales. For this, the authors applied white priming and, subsequently, stochastic speckle patterns with black spray paint on the abaxial seed scale surfaces and investigated the displacement of the scale and the strain on the abaxial surface over time via time-lapse. The two-phase motion could be mapped well by 3D-DIC (**Figure 7A**), showing the synchronous displacement of the lateral scale “flaps” (leading to transverse curvature change and the “flattening” of the scale), followed by longitudinal bending. The strain fields observed are in accordance with this movement behavior, with strong negative strain (up to 6% shrinkage) along the longitudinal scale axis (**Figure 7B**), which becomes more and more pronounced over time and is likely the basis for longitudinal bending, and a strong and negative strain (up to 5% shrinkage) along the transverse axis of the scale likely responsible for the transverse bending (**Figure 7C**).

In summary, the complex movement behavior of pine cone seed scales could be analyzed in detail and the movement sequences explained by highlighting two strain fields with temporal and directional differences, likely dictated by different material properties of the scale. In direct comparison to the motions of uncoated seed scales, the authors note that the desiccation-driven movements of the scales with priming and speckle paint take more time due to a slowing down of the evaporation of water from the scale to the environment. However, the general motion sequences and patterns, which are morphologically predetermined (i.e., nastic), remained unaffected.

Additionally, the authors also developed 4D-printed hygroscopic flaps, inspired from previously published and their own fundamental research on pine cone actuation and deformation. These biomimetic structures were also successfully investigated via 3D-DIC, highlighting the versatility and robustness of the methodology which is applicable for investigations of very variable compliant systems.

#### 4.2.2 The trap shutting and reopening in the Venus flytrap

In contrast to the foregoing example, Sachse et al. (2020) applied 3D-DIC to a living and fast-moving structure, i.e., the snap trap of the Venus flytrap (*Dionaea muscipula*). The Venus flytrap originates from North America and captures small prey animals, predominantly arthropods, for nutrient supply. The ca. 2 cm long traps each consist of two lobes connected by a midrib and snap shut within few 100 ms (Forterre et al., 2005; Poppinga et al., 2016) after prey touches twice the trigger hairs situated in the trap (Scherzer et al., 2019; Saikia et al., 2021). Foregoing detailed analyses (Forterre et al., 2005) revealed that traps are activated by water displacement processes between cells and tissues within each trap lobe, entailing a very rapid snap-through process of the initially concave lobes (as seen from outside the trap) to convex. This elastic snap-buckling acts as a speed boost of the otherwise relatively slow hydraulics at play, so that the trap can overcome fast prey.

**Figure 8:**
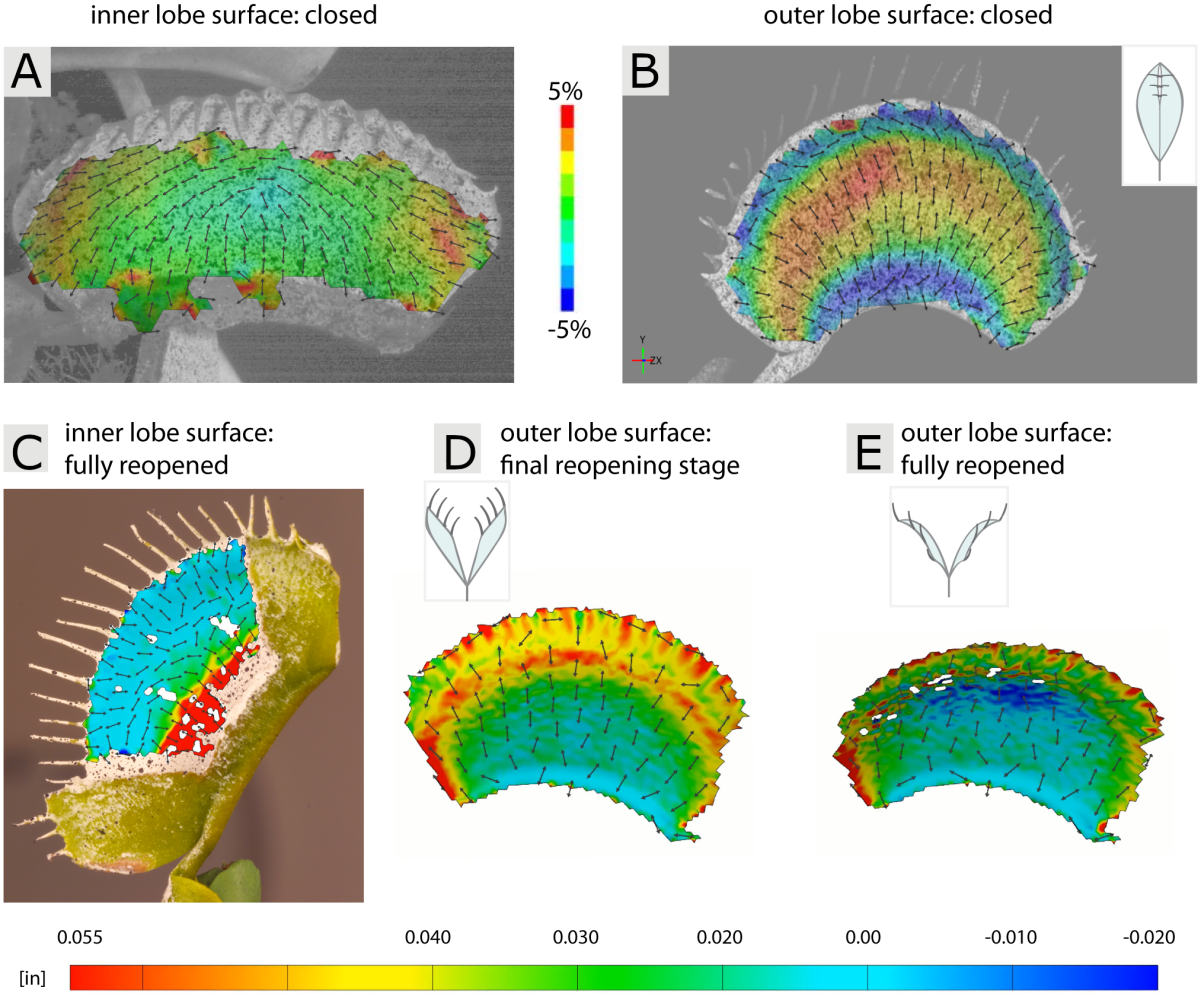
3D-DIC of the inner and outer trap lobe surfaces of the Venus flytrap (*Dionaea muscipula*) during fast snapping, recorded with synchronized highspeed-cameras, and slow reopening, recorded via time-lapse. **(A, B)** Strain distributions during fast snapping in the direction with both higher deformation (denoted as major strain) and lower deformation (minor strain) are shown. **(C)** View on the inner trap lobe surface with strain distribution through a window cut into the opposite lobe in a fully reopened trap. **(D, E)** Strain distribution on the outer lobe surface during two reopening stages. Figure modified after Sachse et al. (2020) and Durak et al. (2022b).

For investigations regarding possible prestress within the open trap, Sachse et al. (2020) conducted series of physiological water stress experiments, 3D-DIC, and FE simulations. For the 3D-DIC analyses, traps were coated with an antiglare spray and, subsequently, a stochastic speckle pattern. Outer as well as inner trap lobe surfaces were investigated (**Figures 8A, B**), the latter by cutting away the opposite lobes. Despite the spray coating and injury inflicted to the trap, the lobes under investigation still behaved naturally and showed typical snapping and reopening movements, highlighting the high applicability of DIC and concomitant procedures to living plant structures. The trap motions were then triggered manually and recorded with two synchronized highspeed-cameras at 1,000 fps and the strain distributions were analyzed. During trap closure, an increase in strain even in time was observed, with the greatest strain on the outer surface (8-10%) at the region positioned centrally and perpendicular to the midrib. Additionally, strain (3-5%) parallel to the midrib at the region where the concave-convex curvature change is most prominent was observed on the outer surface. The midrib and marginal regions of the trap were unaffected. A complex strain evolution and distribution was recorded for the inner surface, where negative and positive strains parallel to the midrib at a central region, and at regions closer to the trap margins were observed. These experimentally gained results were fed into FE simulations, which, together with theoretical swelling/shrinking scenarios of lobe tissue layers and the mentioned physiological experiments, eventually showed that hydraulic prestress is a prerequisite for fast snapping in the Venus flytrap.

After triggering, the trap either reopens when there is no suitable prey inside, or undergoes a stomach formation for digestion of suitable prey (Durak et al., 2022a). In a follow-up study of the fast snapping investigations, Durak et al. (2022b) analyzed the slow reopening of manually triggered (empty) traps, which takes several hours. They used similar methods and procedures as in Sachse et al. (2020), however, two trap size classes were comparatively investigated and time-lapse cinematography applied. Moreover, for analyzing the inner trap lobe surfaces, cutting away the opposite lobe was not an option since in the closed state (the starting point for the reopening investigations) the lobes press against each other. Therefore, windows were cut into the opposite lobe (see **Figure 8C**), with the remaining lobes under investigation still showing typical “reopening” motions. The authors found that traps either reopen smoothly, without any sudden curvature change, or via snap-buckling, and that this behavior stems from a combination of size and slenderness of individual traps. The 3D-DIC examination of the evolving strain patterns on the outer and inner surfaces indicate that both abaxial and adaxial tissue layers are involved in reopening, showing localized shrinking and expansion in specific areas over time (**Figures 8D, E**). Similar to the Sachse et al. (2020) study it could be shown that 3D-DIC is applicable also to small living plant structures, which can undergo deformation in various timescales (fast vs. slow).

### 4.3 DVC for plant biomechanics

The pomelo peel (see section 4.1.3) was not only analyzed by 2D-DIC, but Wang et al. (2018a) tested samples consisting of endocarp and exocarp under stepwise axial compression in a CT scanner. DVC was performed based on one scan in the unloaded state and five scans under compression steps from 10% to 50% strain (see section 3.6). In addition to the segmented trajectories of the vascular bundles and their response to compression, the local volume strain of the different layers could be visualized and quantified. This revealed a non-uniform increase in compression from the endocarp to the exocarp, indicating a cell gradient, and strikingly low strains in the areas around the vascular bundles, illustrating their strong influence on mechanical properties. Keyes et al. (2016) recorded *in-situ* CT images of a root tip of a maize plant growing in soil, and analyzed the related soil displacement using DVC. A growth period of 19 h was recorded at 1 h intervals, with the root showing curved growth with more pronounced soil displacement on the convex curved side.

### 4.4 DIC and DVC for wood and tree analysis

DIC was intensively applied since the early 1990s in wood science. As an alternative to white light speckle photography (Benckert, 1992), Choi et al. (1991) recognized the great potential of DIC for wood and paper characterization and used it for local strain analysis, for example to detect areas of strain outside the elastic range. They analyzed micro-tensile tests in which wood samples were imaged under a microscope using CCD cameras, but were already aware that the technique was equally suitable for analysis of single fibres up to entire timbers and is readily available at relatively low cost. In the following years and decades, DIC was indeed applied to wood analyses ranging from the cell wall (Murata and Masuda, 2001a; Murata and Masuda, 2001b) and its swelling behavior using confocal laser scanning microscopy, over the characterization of isolated wood fibres in micro tensile tests (Mott et al., 1996), to complete beams under four-point bending (Navaratnam et al., 2020) or crack analysis (Dubois et al., 2012). DIC has not only been used to characterize the local mechanical properties of wood, for example stiffness variation along growth rings (Jernkvist and Thuvander, 2001; Ljungdahl et al., 2006; Jeong et al., 2009), fracture behavior (Bjurhager et al., 2008; Ritschel et al., 2013; Ritschel et al., 2014; Xavier et al., 2014), orthotropic properties depending on sample orientation (Jeong et al., 2010a; Jeong and Park, 2016; Henriques et al., 2022) “or” Poisson`s ratio (Zink et al., 1995; Zink et al., 1997; Jeong et al., 2010b). It has also been used to analyze strains along wood knots (Oscarsson et al., 2012), around multiple bold-wood connections (Stelmokas et al., 1997), at timber finger joints (Konnerth et al., 2006), around crack tips (Samarasinghe and Kulasiri, 2004), in steel-timber joints (Sjödin et al., 2006), wood adhesive bonds (Serrano and Enquist, 2005) “or” in laminated composites (Muszyński et al., 2000). In addition to digital cameras or digitized photographs (Zink et al., 1995), optical microscope images (Jernkvist and Thuvander, 2001) or cross-sections from CT scans (Danvind, 2005; Watanabe et al., 2012) were also used as image source for 2D-DIC analysis. The results of the several approaches were compared with, or used as a basis for, the results of FE simulations (Serrano and Enquist, 2005; Sjödin et al., 2006; Muszyński and Launey, 2010; Oscarsson et al., 2012). Comparative experiments with mechanical extensometers showed that DIC has the advantage of being able to analyze wood samples of different thicknesses, whereas extensometers damage samples that are too thin and can cause slippage artefacts in samples that are too thick (Jeong et al., 2010b). Valla et al. (2011) also compared DIC with electronic speckle pattern inferometry (ESPI; see also Müller et al., 2005), another optical, non-contact technique for measuring full-field surface displacements and strains. ESPI involves illuminating a surface with two coherent laser beams from two different directions and recording the reflected light waves, resulting in a speckle interferogram. Displacements on the surface result in phase differences that can be localized with high spatial accuracy. However, the major advantages of DIC over ESPI are the rather easy experimental setup, the ability to record experiments continuously, the versatility and flexibility of the method, and the moderate cost for standard applications (Valla et al., 2011).

For more than a decade, DVC has been used alongside DIC in the analysis of wood structures. For example, Forsberg et al. (2008) performed synchrotron CT (SRμCT) imaging of wood under three-point bending. The 1.57 × 3.42 × 0.75 mm³ Scots pine bar-shaped wood sample was scanned in the unloaded state and under three loading conditions with a resolution of approximately 2 µm. In these *in-situ* tests, the influence of small cracks in the specimen and the support and force application locations for the bending test could be visualized in a high resolution and localized manner as 2D and 3D strain maps. In a follow-up paper, Forsberg et al. (2010) elaborate on the methodology and emphasize that wood, with its complex cellular structure, is a very suitable material for DVC experiments. The importance of this natural material texture (not only on the surface but throughout the volume) for successful DVC analysis is further emphasized in the review by Bay (2008). Further *in-situ* mechanical testing of wood under incremental compression loads was carried out on wood-based fiberboards coupled with µCT scanning (Tran et al., 2013). These showed that a decrease in porosity was measurable up to a compression level of 30%, and that fiber failure only occurred at higher compression levels. Ching et al. (2018) also used µCT scans to measure 3D strain under shear stress in wood adhesive bond samples, which were tagged with iodine for contrast enhancement. They revealed that changes in the microstructure (wood rays and resin channels) have a marked influence on the local strain distribution. Derome et al. (2011) exploited the natural swelling and shrinkage behavior of Norway spruce wood at defined moisture levels to perform 3D strain measurements using SRμCT. Swelling and shrinkage strains were found to be more pronounced in latewood than in earlywood, with latewood cells retaining their initial shape.

**Figure 9:**
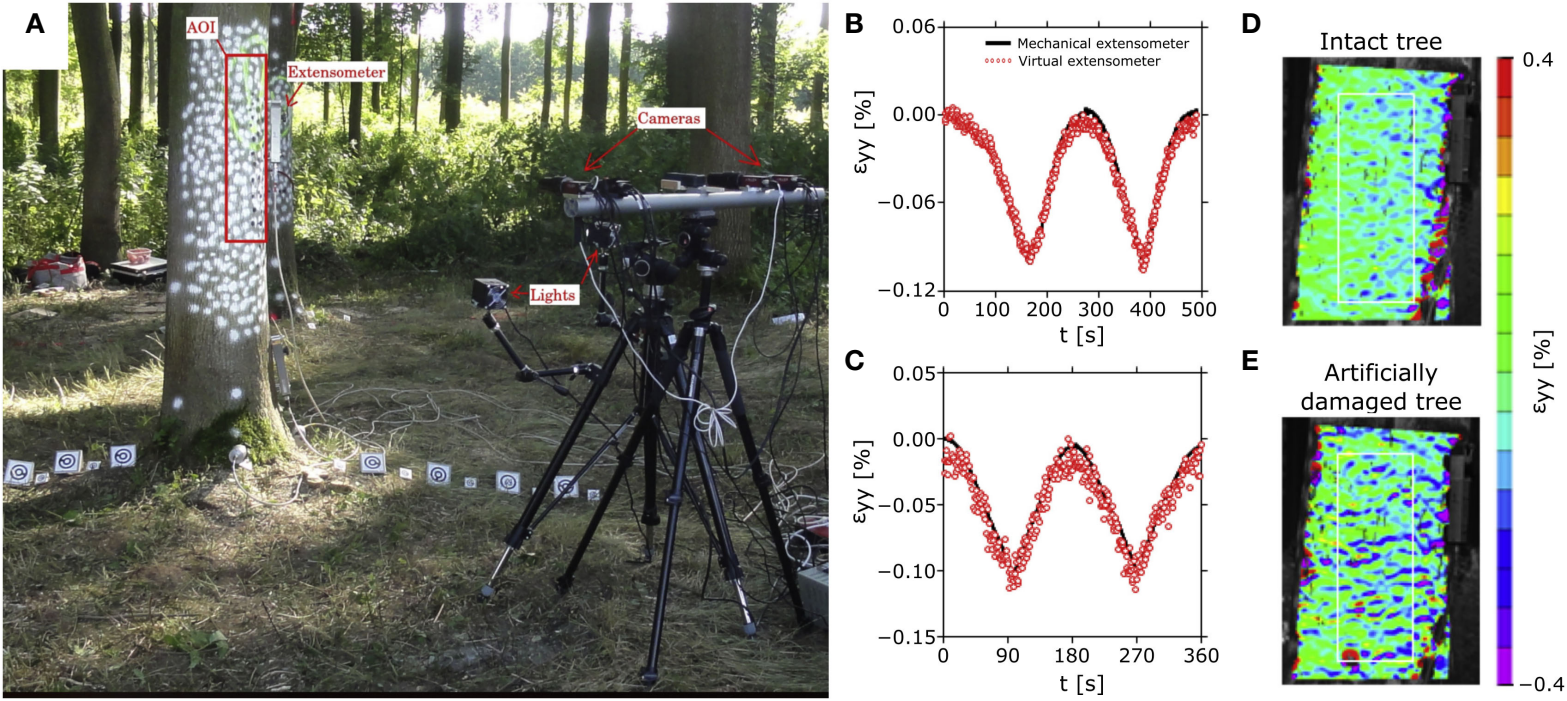
3D-DIC analysis of a tree trunk with mechanical extensometers for comparative measurement of local strains. **(A)** Measurement setup of the field experiment on an ash tree (*Fraxinus excelsior*) trunk for comparison between non-contact 3D-DIC and an invasive mechanical extensometer. The strains in the vertical direction ϵ_yy_ caused by mechanical pulling on the tree at a height of 15 m are shown for two trees **(B, C)** for comparison between measurements with mechanical extensometers and virtual extensometers (3D-DIC). In addition, the resulting strain patterns were compared between an intact tree trunk **(D)** and the same area after sawing out a rectangular piece of the tree (25⨯15⨯17 cm³) on the back side of the tree **(E)**. Figure modified from Tippner et al. (2019).

In addition to dissected wood samples, DIC was also used to analyze intact trees (**Figure 9**). For a mechanical trunk analysis, they trunks were subjected to bending stress by pulling. Sebera et al. (2014) applied 3D-DIC to analyze bark displacement and deformation of Persian walnut trees, with gaps in surface detection due to the complex bark surface geometry. A similar study on the Turkish hazel analyzed the influence of the bark by carrying out experiments with bark and without bark (Sebera et al., 2016). Measurements on the xylem showed complete surface detection and indicated that the bark absorbed a significant proportion of the resulting strains. No effect of strain measurements on samples with or without bark was found for young red oak branches, but the bark tested was much less pronounced at thicknesses less than 2 mm (Dahle, 2017). Tippner et al. (2019) used 3D-DIC imaging to non-invasively detect defects in tree branches and roots (**Figures 9D, E**). Applying 3D-DIC on root-trunk transition zones, Beezley et al. (2020) analyzed how forces are transmitted and distributed through pin oak trees, contributing to the risk assessment of damaged trees. Eckenrode (2017) studied branch attachments and used 3D-DIC to gain a better understanding of how and why branch failure occurs, resulting in publications on branch union load distribution in oak (Eckenrode et al., 2022) and red maple (Dahle et al., 2022) trees.

Traditionally, semi-destructive and locally limited extensometers have been used for strain analysis of trees. Compared to the optical DIC technique, they showed comparable results (Tippner et al., 2019; **Figures 9B, C**) or a good agreement of the strain curves with slightly lower strain values in the DIC measurements (Sebera et al., 2014; Sebera et al., 2016). Because of its full-field measurements, the DIC method can replace several hundred extensometers and is also recommended for repeated comparative measurements over several years due to its non-destructive nature (Dahle, 2017).

## 5 Summary and outlook

DIC is a non-contact, optical method for analyzing displacements and strains that is readily applicable to plant scientists working on plant biomechanics and functional morphology, morphogenesis, plant movements, and potentially many other areas. The examples presented have shown that DIC can help to answer complex questions in a complementary way to classical and modern computer-based methods. In addition to the basic non-contact analysis method, invasive sample preparation may be required, depending on the intended application. However, in the examples shown from the field of plant movements (slow passive swelling and shrinking of dead cell assemblies in pine cones, fast and slow snapping of living traps of carnivorous plants), the interventions were too minor to cause notable disturbances or deviations from the natural behavior, i.e., movement patterns. Here, 3D-DIC in particular has emerged as a powerful tool to quantify temporally and spatially complex deformation processes driven by various actuation principles in high resolution. In addition to the ever-expanding applications of 2D-DIC and 3D-DIC, its sister technique, DVC, promises further opportunities to characterize plant structures by providing complementary information from full volume displacement and deformation analysis during *in-situ* experiments.

## Author contributions

MM: Conceptualization, Visualization, Writing – original draft. SP: Conceptualization, Visualization, Writing – original draft.
